# Investigation on the Microstructure of ECAP-Processed Iron-Aluminium Alloys

**DOI:** 10.3390/ma14010219

**Published:** 2021-01-05

**Authors:** Bernd-Arno Behrens, Kai Brunotte, Tom Petersen, Roman Relge

**Affiliations:** Institute of Forming Technology and Machines, Leibniz Universität Hannover, An der Universität 2, 30823 Garbsen, Germany; behrens@ifum.uni-hannover.de (B.-A.B.); brunotte@ifum.uni-hannover.de (K.B.); petersen@ifum.uni-hannover.de (T.P.)

**Keywords:** iron-aluminium alloys, microstructure, equal channel angular pressing (ECAP), hot forming, bulk forming

## Abstract

The present work deals with adjusting a fine-grained microstructure in iron-rich iron-aluminium alloys using the ECAP-process (Equal Channel Angular Pressing). Due to the limited formability of Fe-Al alloys with increased aluminium content, high forming temperatures and low forming speeds are required. Therefore, tool temperatures above 1100 °C are permanently needed to prevent cooling of the work pieces, which makes the design of the ECAP-process challenging. For the investigation, the Fe-Al work pieces were heated to the respective hot forming temperature in a chamber furnace and then formed in the ECAP tool at a constant punch speed of 5 mm/s. Besides the chemical composition (Fe9Al, Fe28Al and Fe38Al (at.%—Al)), the influences of a subsequent heat treatment and the holding time on the microstructure development were investigated. For this purpose, the average grain size of the microstructure was measured using the AGI (Average Grain Intercept) method and correlated with the aforementioned parameters. The results show that no significant grain refinement could be achieved with the parameters used, which is largely due to the high forming temperature significantly promoting grain growth. The holding times in the examined area do not have any influence on the grain refinement.

## 1. Introduction

Iron-aluminium alloys have been researched and developed for many years. The particular interest lies in the many advantages of these alloys, which make them very versatile. Because of their corrosion resistance in an oxygen- and sulphur-containing environment, their high melting point, and their high strength at low density (up to 25% weight reduction with Fe38Al (at.%—Al) compared to steel), they are used, for example, as high temperature materials in the aerospace industry [1]. Depending on the composition, iron-aluminium alloys are also used for other industrial applications, e.g., brake disks for windmills and trucks, filter systems in refineries and fossil power plants, and transfer rollers for hot-rolled steel strips, as well as ethylene crackers and air baffles to burn high-sulphur coal [2,3]. Thanks to the low price, the good availability, and recyclability of iron and aluminium, iron-aluminium alloys are interesting from an economic point of view for industrial use [4]. However, due to the very low ductility at room temperature caused by hydrogen embrittlement in a humid environment (elongation at break well below 5%) and the rapid drop in strength at temperatures above 600 °C, iron-aluminium alloys have long been neglected in structural applications [5]. The current research interest is to improve the ductility at room temperature and to increase the formability in the hot forming area.

Iron-aluminium alloys count among the intermetallic compounds. As a rule, this type of connection has completely different properties compared to its metallic alloy components. Depending on mixing ratio and temperature, the iron-aluminium alloys can exist in three essential intermetallic phases A2, B2, and D03 on the iron-rich side [1,4,5,6].

The decreasing ductility with increasing aluminium content is an exclusion criterion for the large-scale use of iron-aluminium alloys. Many studies deal with the increase in ductility and creep strength by alloying with chromium, molybdenum, and other additives. As a result, elongations at break of up to 7% could be achieved for the Fe3Al-phase of iron-aluminium alloys, but this value is still below the required 10% to make it suitable for structural component production [5,6].

Grain refinement is one of the few strengthening mechanisms that favours both strength and ductility in the micrometre-regime [7], but this effect is inversed in the lower nanometre-regime [8]. Hall et al. demonstrated that a decreasing grain diameter of a metal structure causes an increase in yield strength and set the Hall–Petch equation for this [9,10]. Grain refinement also increases the grain boundaries. The interaction of the grain boundary atoms is believed to contribute to the ductility of metals [7]. An increase in grain interfaces, thus, also causes an increase in ductility. In addition, studies have shown that grain boundaries are an obstacle to the movement of hydrogen, which could reduce the danger of brittle fracture due to hydrogen embrittlement of Fe-Al alloys [11].

Morris et al. summarised research results on the influence of grain size on the ductility of binary iron-aluminium alloys produced by powder metallurgy [7]. As the grain diameter decreases, the ductility increases exponentially. In this way, elongations at break of up to 7% could be achieved in the single-digit micrometre range.

In contrast to the ultra-fine-grained structure produced by Morris et al. [7], this work deals with the setting of a fine-grained structure by means of hot massive forming. For this, the results of previous works were taken into account, where iron-aluminium alloys in the as-cast state were examined for their mechanical properties [12], then for forming behaviour [13,14], and finally, the mechanical properties after forming [15,16]. The toughness of components made by powder metallurgy is generally lower than that of components produced by casting and forming due to the increased porosity caused by the manufacturing process. As a result, an improved elongation at break from the casting and forming of ultra-fine-grained iron-aluminium alloys can be expected.

**Figure 1:**
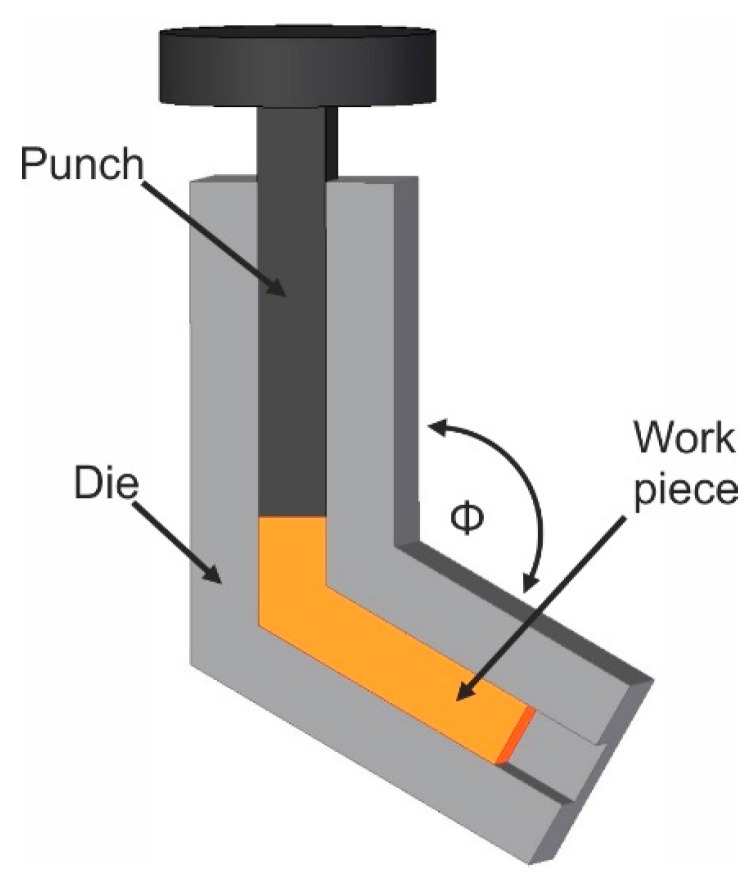
Principle sketch of the ECAP-process (Equal Channel Angular Pressing).

A forming process that enables the setting of an ultra-fine-grained structure is the ECAP-process (Equal Channel Angular Pressing) [17]. In this process, a work piece is pressed with a punch through two channels with the same cross-section, which are arranged at an angle to each other (Figure 1). Depending on the degree of deformation, this angle can be between *Φ* = 90° and 120°.

The process leads to shear stress on the structure and, thus, to grain refinement [17]. The special feature of this forming process is the possibility to perform several passes on the same work piece, as the cross-section of the work piece does not change. The true-strain add up with each pass and the microstructure can be further refined. In the literature, forming using the ECAP-process was carried out predominantly at lower temperatures using ductile materials such as aluminium alloys [18]. The forming of intermetallic alloys, such as titanium alloys, was investigated at higher temperatures, due to the low ductility [19].

## 2. Materials and Methods

Before ECAP forming, the samples had to be prepared as follows. Representative of the three iron-rich phases, samples with the nominal compositions Fe9Al, Fe28Al, and Fe38Al (at.%—Al) were selected for the property investigations and tests on the forming behaviour of iron-aluminium alloys. These binary iron-aluminium alloys were cast to 50 kg ingots, and then wire-cut to sample sizes of 140 mm × 140 mm × 20 mm using wire EDM (electrical discharge machining). Subsequently, incremental hot forming was used for reshaping at different temperatures, Fe9Al at 1250 °C, Fe28Al at 1150 °C, and Fe38Al at 1100 °C, with a punch speed of 30 mm/s; these forming parameters were determined by Huskic [16]. These parameters shape the finest grain sizes possible through the conventional forming of iron-rich iron-aluminium alloys for these nominal compositions without cracks. In addition, forming removes the casting defects such as internal pores that are negatively affecting the material properties of casts. After incremental hot forming and cooling, the samples were heat treated at 750 °C in order to homogenize the microstructure and increase the deformability of the forged specimens. Sikka et al. [5] found that a maximum increase in ductility of approx. 15 to 20% in Fe_3_Al alloys with an Al content of 28 at.% can be achieved after a thermo-mechanical process and a heat treatment of one hour at temperatures of 750 °C. Huskic’s [16] heat treatment at the same temperature gave similar results for the three examined alloys. In the process, most of the formed grains recrystallised, which led to a grain refinement in comparison to the cast structure and an improvement in the deformability. The holding times determined by Huskic [16] (1 h Fe28Al and Fe38Al; 2 h Fe9Al) were used for the heat treatment. After incremental hot forming and heat treatment, the samples were wire-cut by means of wire EDM from a blank geometry of 40 mm × 40 mm × 240 mm to the required ECAP sample size of 14 mm × 14 mm × 75 mm.

**Figure 2:**
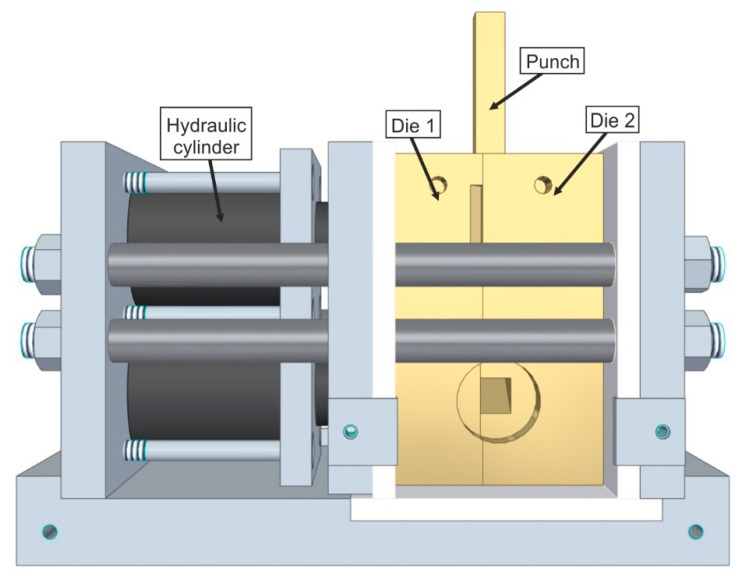
Design of the equal channel angular pressing tool.

The samples and the ECAP tool, consisting of Inconel 718, were heated to 1100 °C for 15 min (samples) and 45 min (tool) in a chamber furnace. In order not to lose any heat transfer, the samples were placed in the ECAP tool in the chamber furnace, and then were formed using the ECAP method with a constant punch speed of 5 mm/s (Figure 2). The tool consisted of two die halves that were attached with the hydraulic cylinders in order to withstand lateral forces. Thus, a quick removal of the sample could be guaranteed. The angle *Φ* of the ECAP die was 120°, which corresponds to a true-strain of *φ* = 0.7. This large angle ensures that the brittle iron-aluminium alloys are stressed as little as possible during the forming process. The formability of Fe28Al and Fe38Al is limited even at high temperatures [16].

After forming, the samples were cooled to room temperature in still air and then heat-treated at 750 °C with variable holding times (up to 90 min in 15 min steps) in the chamber furnace. In addition to the influence of the deformation on microstructure development through ECAP forming, the influences of a subsequent heat treatment were investigated. For this purpose, the average grain size of the microstructure was measured using the AGI (Average Grain Intercept) method. A total of 6 lines of the same length (3 horizontal and 3 vertical) were laid over the entire microstructure at the same distance and the cut grains per line were counted. The average grain sizes and standard deviations result from these 6 lines. The degree of recrystallisation, which represents the percentage of the area of the recrystallised grains versus the area of the deformed grains, was determined by visual inspection. For this purpose, the areas of the recrystallised grains were determined with a conventional drawing program and were divided by the total area. Statements can, thus, be made about whether and how the holding period of the heat treatment influences grain growth. In order to examine the influences of the ECAP forming on the parameters carried out as well as the subsequent heat treatment and its holding time on the grain size development, a comparison was made with the grain sizes of the cast structure determined by Huskic [16] and the formed plus heat-treated samples. The reshaped and heat-treated structure corresponds to the incrementally reshaped and heat-treated samples that were carried out in this work. In order to visualize the structure, the microstructural examination by means of light microscopy is followed by etching in a solution of 40% HCl, 40% HNO_3_, and 20% H_2_O. The microstructural images were recorded digitally by means of a stereo microscope from Leica Mikrosysteme GmbH (M series, Wetzlar, Hesse, Germany).

## 3. Results

### 3.1. Fe9Al

**Figure 3:**
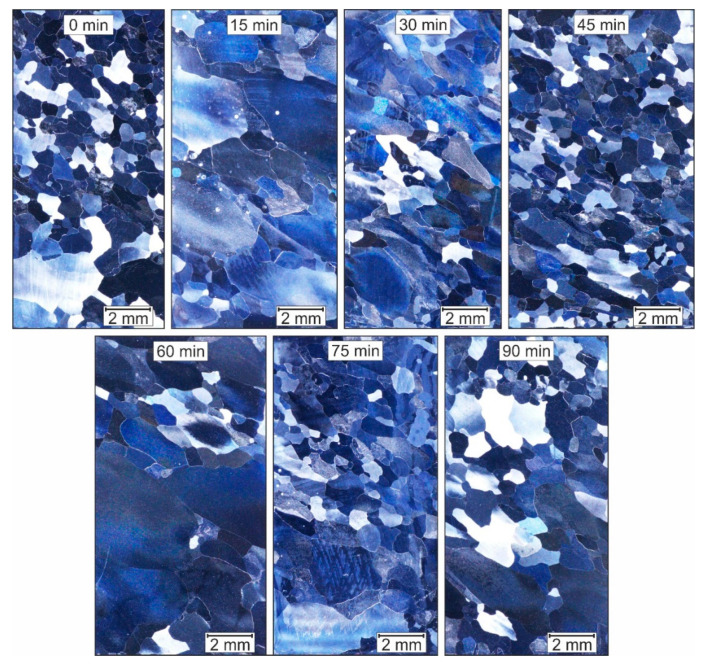
Fe9Al after one ECAP Pass and heat-treated at 750 °C with different holding times.

Figure 3 shows the microstructure images of Fe9Al, which were once reshaped in the ECAP and then heat-treated at 750 °C for different holding times. Table 1 summarizes the calculated mean grain diameter and the degree of recrystallisation.

It can be seen that even before the heat treatment (0 min), a fine structure of around 500 µm has developed and a high degree of recrystallisation is present. Samples held for 30, 45, and 75 min show similar values. However, the samples held for 15 and 60 min, show a low recrystallisation rate and, therefore, have a mean grain diameter that is twice as large. Shear bands that can arise during the ECAP-process are not observed. Compared to the cast structure, the structure according to the ECAP has a finer grain (Table 1). However, taking into account the results of the Huskic [16] structure after conventional upsetting and identical heat treatment, no significant grain refinement can be observed. The desired ultra-fine-grain size through the ECAP process was not achieved. 

### 3.2. Fe28Al

**Figure 4:**
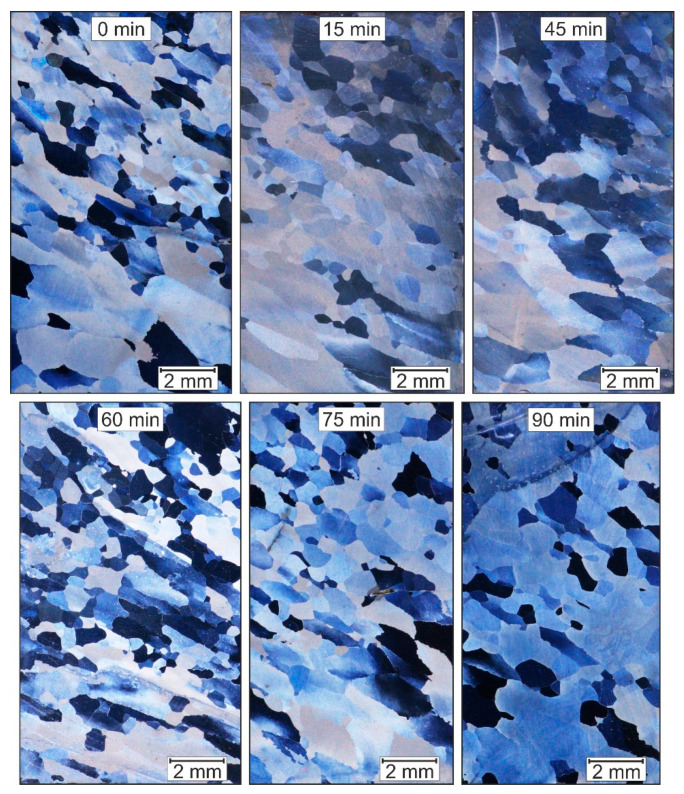
Fe28Al after one ECAP Pass and being heat-treated at 750 °C with different recovery times.

Figure 4 shows the microstructure images of Fe28Al, which was reshaped once in an ECAP-process. The heat treatment also took place at 750 °C and different holding times of up to 90 min in 15-min steps. The sample with a holding time of 30 min was not considered due to incorrect preparation. Table 2 summarizes the calculated mean grain diameter and the degree of recrystallisation.

The size of the grains is more homogeneously distributed compared to Fe9Al. However, there is a tendency towards more deformed grains in the lower region. The middle and upper area is dominated by finer, recrystallised grains. The holding time of the subsequent heat treatment shows no influence on grain growth or a purely clear indication of static recrystallisation.

The mean grain size after the ECAP-process 1 h heat treatment is smaller than in the cast state, but not much finer than the results of Huskic after conventional upsetting and identical heat treatment. However, after the ECAP-process, the grade of recrystallisation is higher than after conventional forming.

### 3.3. Fe38Al

**Figure 5:**
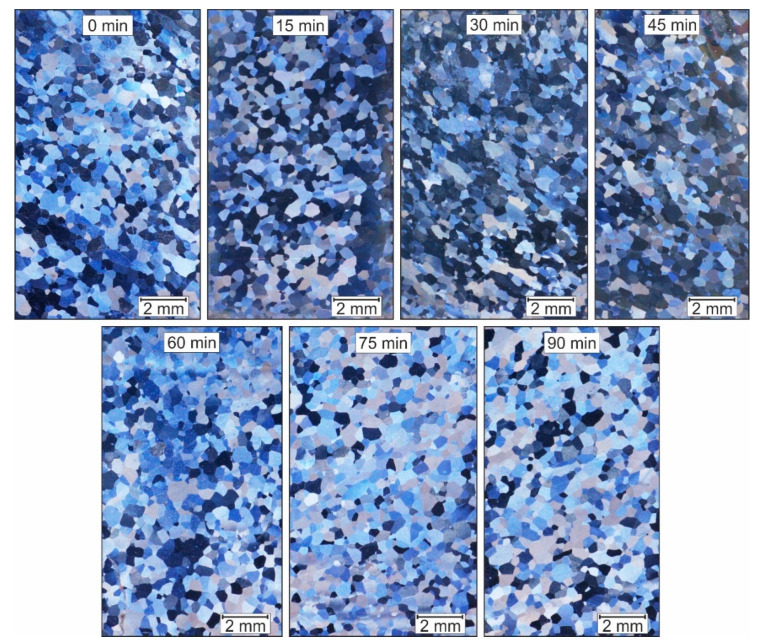
Fe38Al after one ECAP Pass and being heat-treated at 750 °C for different recovery times.

As shown in Figure 5, the microstructure of Fe38Al, which was reshaped once in the ECAP, and then heat-treated at 750 °C for different holding times, is fully recrystallised. Fe38Al samples also reached the same results in conventional upsetting [16].

Regardless of the duration of heat treatment after the ECAP-process, the mean grain diameters are almost identical (Table 3). This means that neither the heat treatment temperature nor the duration of heat conversion has an influence on grain growth or grain refinement due to static recrystallisation.

The comparison with the mean grain sizes from the as-cast state shows a clear grain refinement. Compared to the compressed samples, however, no improvement has been achieved here either. The grain sizes are even identical for both forming processes.

## 4. Discussion

As can be seen from the results, the ECAP-process does not result in any (Fe28Al and Fe38Al) or significant refinement (Fe9Al) of the grain compared to the previous forming step, the incremental forming of casts. The desired ultra-fine grain size through the ECAP process was not achieved with these forming parameters. This is due to the low true-strain caused by the wide die angle *Φ* and to the high forming temperature reducing the shear stresses through dynamic recrystallisation. Upsetting tests of iron-aluminium alloys at lower forming temperatures (900 °C) and subsequent recovery have resulted in a finer grain than at 1100 °C. Lowering the temperature led to cracks in the iron-aluminium samples and resulting tool failure at 800 °C. The formation of shear bands is obviously prevented by the large die angle *Φ* and the dynamic recrystallisation caused by the high forming temperature.

### 4.1. Fe9Al

**Figure 6:**
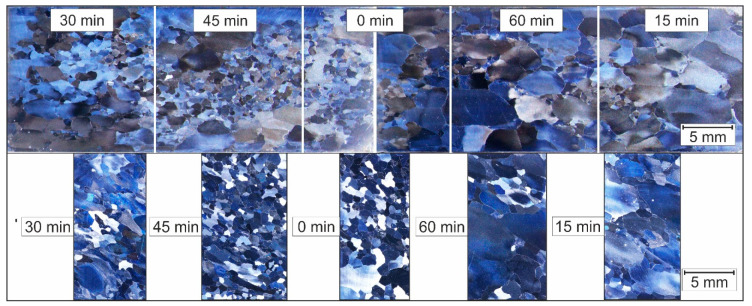
The forming history of the microstructure of Fe9Al. Above: microstructure and sampling point before ECAP-process (left half fine grain, right half coarse grain); below: after one ECAP pass and heat-treated at 750 °C with different recovery times.

At first sight, the samples with a recovery time of 15 and 60 min show contradicting results with regard to the other samples. The low degree of recrystallisation is due to two reasons, the local position of the sampling as well as the deformation history of the previous incremental deformation. Figure 6 shows the sampling before the ECAP-process. It can be seen that the point from which the samples for 15 and 60 min were taken has experienced a lower degree of shaping during incremental forming, which leads to an inhomogeneous distribution of the structure.

### 4.2. Fe28Al

The ECAP-process of Fe28Al at 1100 °C leads to a halving of the grain size, but lower values than those of Huskic [16] after conventional forming are not reached. However, the degree of recrystallisation after the ECAP-process is higher and, thus, the homogenisation of the microstructure is as well.

**Figure 7:**
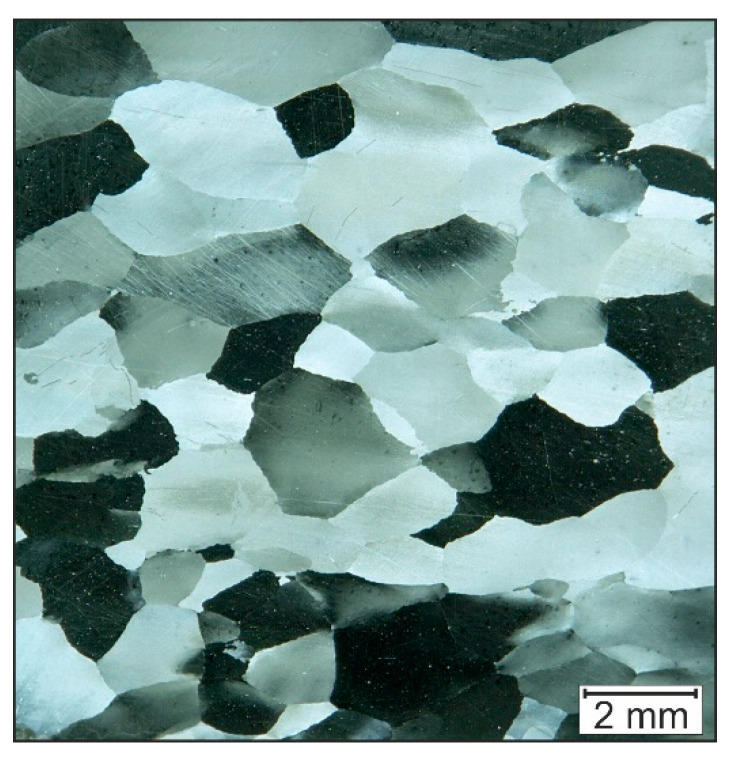
Microstructure of Fe28Al before ECAP forming.

The true-strain has a strong influence on the dynamic recrystallisation of Fe28Al [16]. In the designed ECAP-process, the global degree of deformation is *φ* = 0.7, which is lower compared to the compressed samples by Huskic (*φ* = 1.2). Figure 7 shows the microstructure of Fe28Al after incremental forming, before the ECAP-process. The mean grain size is 1100 µm. It can thus be concluded that the ECAP-process resulted in a fine-graining of the microstructure. Further forming by ECAP could result in further grain refinement.

### 4.3. Fe38Al

**Figure 8:**
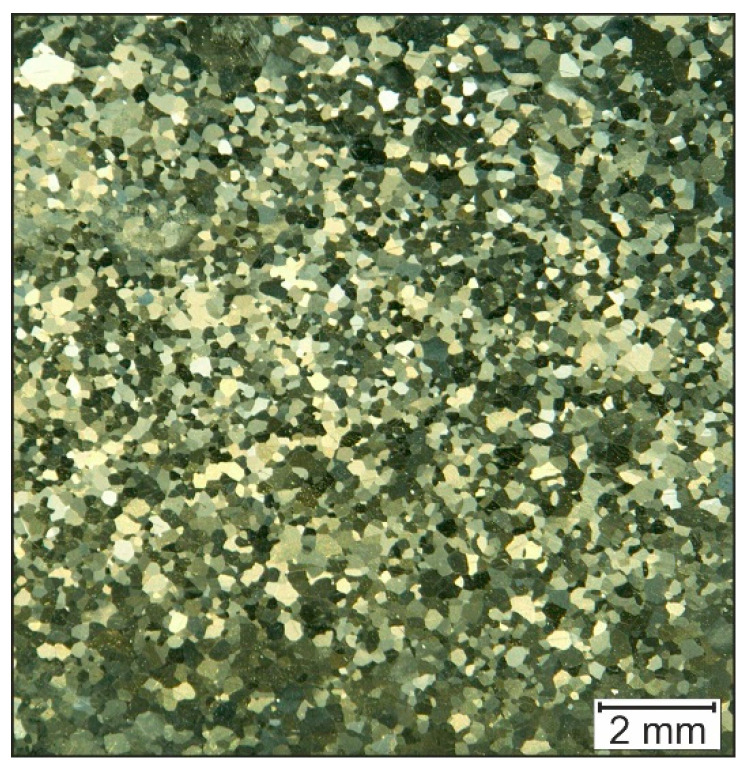
Microstructure of Fe38Al before ECAP forming.

The ECAP-process of Fe38Al also did not cause any grain refinement of the structure. If the microstructure of the alloy is examined after incremental forming (Figure 8), an even finer grain than achieved with ECAP can be seen (150 µm). This is probably due to the fact that the incremental forming of Fe38Al at 1100 °C could not be carried out completely isothermally since the work piece cools down to approximately 900 °C. The upsetting tests by Huskic revealed that the structure of Fe38Al shows a finer development at lower temperatures, which explains the finer grain structure reached in incremental forming. This means that the higher forming temperature in the ECAP process led to a grain size increase compared to incremental forming.

## 5. Conclusions

This work shows that the processing of iron-aluminium alloys by ECAP with the specified forming parameters does not lead to any significant grain refinement. Here the ECAP forming temperature is the greatest influencing factor for grain refinement and recrystallisation. The typical shearing behaviour of the grains, which in the ECAP-process of cold-formed metals contributes to grain refinement, is not recognizable at the temperatures investigated because of the wide angle *Φ* and high forming temperature.

- Fe9Al has not completely recrystallised after ECAP forming and the distribution of the grain sizes is inhomogeneous due to the forming history.
- Fe28Al also has not completely recrystallised, but has a homogenised microstructure compared to Fe9Al regardless of the heat treatment. The mean grain size is also finer than the microstructure before the ECAP-process. A lowering of the deformation temperature of Fe28Al would, however, lead to cracks due to the low deformability.
- The ECAP-process of Fe38Al has led to a doubling of the mean grain size. A grain refinement of Fe38Al is not possible with the forming parameters applied. Similar to Fe28Al, a lowering of the forming temperature leads to cracks in the samples. A steeper angle is also not viable due to cracking. Shortening the heating time and immediate cooling after reshaping could prevent grain growth of the finely recrystallised structure. However, it is doubtful whether it will be more finely expressed than the structure after incremental forming.

## Figures and Tables

**Table 1:** Comparison between Fe9Al formed through ECAP plus being heat-treated at 750 °C for different holding times, and the results of cast/bulk-formed Fe9Al at 1100 °C with 2 h of heat treatment (ht) [16].

Holding Time of ht in min.	0	15	30	45	60	75	90	As Cast ^1^	Formed + ht 2h ^1^
Average grain size in µm	500 ± 60	1010 ± 150	570 ± 90	440 ± 50	1290 ± 210	510 ± 70	770 ± 80	~2000	640
Grade of recrystallisation in %	74	29	59	77	36	65	59	0	50

^1^ Huskic [16].

**Table 2:** Comparison between Fe28Al formed through ECAP plus being heat-treated at 750 °C for different holding times, and the results of cast/bulk-formed Fe28Al at 1100 °C with 1 h of heat treatment (ht) [16].

Holding Time of ht in min.	0	15	45	60	75	90	As Cast ^1^	Formed + ht 1h ^1^
Average grain size in µm	580 ± 80	670 ± 90	780 ± 80	420 ± 40	620 ± 70	810 ± 60	~2000	530
Grade of recrystallisation in %	66	70	67	71	78	84	0	50

^1^ Huskic [16].

**Table 3:** Comparison between Fe38Al formed through ECAP plus being heat-treated at 750 °C for different holding times, and the results of cast/bulk-formed Fe38Al at 1100 °C with 1 h of heat treatment (ht) [16].

Holding Time of ht in min.	0	15	30	45	60	75	90	As Cast ^1^	Formed + ht 1h ^1^
Average grain size in µm	330 ± 30	290 ± 40	310 ± 40	300 ± 30	340 ± 30	330 ± 40	320 ± 30	~2000	320
Grade of recrystallisation in %	100	100	100	100	100	100	100	0	100

^1^ Huskic [16].

## Data Availability

The data presented in this study are available on request from the corresponding author. The data are not publicly available due to privacy.
